# Luminal-Applied Flagellin Is Internalized by Polarized Intestinal Epithelial Cells and Elicits Immune Responses via the TLR5 Dependent Mechanism

**DOI:** 10.1371/journal.pone.0024869

**Published:** 2011-09-15

**Authors:** Tonyia Eaves-Pyles, Heng-Fu Bu, Xiao-di Tan, Yingzi Cong, Jignesh Patel, Robert A. Davey, Jane E. Strasser

**Affiliations:** 1 Department of Microbiology and Immunology, University of Texas Medical Branch, Galveston, Texas, United States of America; 2 Center for Intestinal and Liver Inflammation Research, Children’s Memorial Research Center, Feinberg School of Medicine, Northwestern University, Chicago, Illinois, United States of America; 3 University of Cincinnati Medical School, Cincinnati, Ohio, United States of America; Albany Medical College, United States of America

## Abstract

Bacteria release flagellin that elicits innate responses via Toll-like receptor 5 (TLR5). Here, we investigated the fate of apically administrated full length flagellin from virulent and avirulent bacteria, along with truncated recombinant flagellin proteins in intestinal epithelial cells and cellular responses. Flagellin was internalized by intestinal epithelial cell (IEC) monolayers of IEC-18. Additionally, apically applied flagellin was internalized by polarized human Caco-2BBe and T-84 cells in a TLR5 dependent mechanism. More, flagellin exposure did not affect the integrity of intestinal monolayers. With immunofluorescent staining, internalized flagellin was detected in both early endosomes as well as lysosomes. We found that apical exposure of polarized Caco-2BBe and T-84 to flagellin from purified *Salmonella*, *Escherichia coli* O83:H1 (isolate from Crohn’s lesion) or avirulent *E. coli* K12 induced comparable levels of basolateral IL-8 secretion. A recombinant protein representing the conserved amino (N) and carboxyl (C) domains (D) of the flagellin protein (ND1/2ECHCD2/1) induced IL-8 secretion from IEC similar to levels elicited by full-length flagellins. However, a recombinant flagellin protein containing only the D3 hypervariable region elicited no IL-8 secretion in both cell lines compared to un-stimulated controls. Silencing or blocking TLR5 in Caco-2BBe cells resulted in a lack of flagellin internalization and decreased IL-8 secretion. Furthermore, apical exposure to flagellin stimulated transepithelial migration of neutrophils and dendritic cells. The novel findings in this study show that luminal-applied flagellin is internalized by normal IEC via TLR5 and co-localizes to endosomal and lysosomal compartments where it is likely degraded as flagellin was not detected on the basolateral side of IEC cultures.

## Introduction

Various species of Gram-negative [1] and some Gram-positive [2] bacteria have thousands of motile hair-like structure called flagella extending from their outer membranes. Flagella structures enable the bacteria to move through its aqueous environment, and attach to and invade host cells [I, 3–5]. Flagellin is the primary protein component that forms the flagellar structure. The flagellin protein folds to form a hairpin arrangement that has been divided into three domains [6]–[9]. The folding of the flagellin protein is such that domains 1 (D1) and 2 (D2) are discontinuous and are formed when residues in the amino terminus (N) and carboxyl terminus (C) are juxtaposed by the hairpin structure [6]–[8]. The middle hypervariable domain (D3) loops out of the hairpin [6]–[8]. Thus, the linear arrangement of the domains is amino domain (ND)1, ND2, D3, carboxyl domain (CD)2 and CD1.

Although flagellin is assembled into flagellum, uncapping of the structure or leakage can lead to the release of flagellin monomers [1], [6], [8], [10]–[12]. A number of circumstances can cause flagellin monomers to be released from the flagella structure. There is a deliberate ejection of the flagellum by *Caulobacter crescentus* when it is no longer necessary for the bacterial life cycle [10]. Alternatively, the shearing of flagella from the bacterial surface can occur via host factors and environmental circumstances such as host proteases, pH, temperature and/or bile salts [12]. Physical forces and chemical factors at the sites of bacterial infection can also shear flagella from bacteria and cause the liberation of flagellin monomers into the surrounding environment where they bind to their receptor, TLR5 [12]. The binding of flagellin to TLR5 is localized to the amino- and carboxyl-conserved regions of the flagellin protein [13]. The structure of the intact flagellum is such that these conserved flagellin protein regions are buried within the flagella filament thus not accessible to Toll-like receptor 5 [TLR5; 12,14]. Therefore, the intact flagella structure is not able to stimulate TLR5 [14], [15] but when the structure depolymerizes flagellin monomers are liberated, exposing the amino- and carboxyl-conserved regions so as to constitute binding to and stimulation of TLR5 [13], [15].

We reported previously that the conserved amino and carboxyl domains of the flagellin protein were responsible for inducing inflammatory responses in intestinal epithelial cells (IEC) and human monocytes via the NF-kB signaling pathway [13]. We reported that a chimeric flagellin fusion protein was generated and evaluated which contained the ND1 and ND2 and CD2 and CD1 domains separated by an *E. coli* hinge element (ECH). The ECH replaced the flagellin D3 region and created a protein hairpin that brought the N and C domains into juxtaposition which we designated ND1/2ECHCD2/1 [13]. The combination of the conserved flagellin regions was later found to bind and activation TLR5 [15]. Therefore, because TLRs recognize conserved products common to both pathogen and commensal bacteria, it is reasonable to hypothesize that either variety of bacteria can stimulate innate immune responses. It has been shown that flagellin from a commensal strain of E.coli triggered IL-8 secretion [16] and CCL-20 [17] expression in mouse and human IEC via TLR5. However, IEC responses to luminal flagellin as well as the fate of flagellin remain undefined.

Our previous findings showed that in healthy mice, luminal application of purified flagellin induced rapid migration of subepithelial dendritic cells strategically positioning them for antigen capture [18]. Further, our additional findings showed that peptidoglycan was taken up in IEC from the AP side, which was mediated through TLR2 [19]. Therefore, we hypothesized that flagellin may also be internalized by IEC following binding to apically expressed TRL5. In this current study we examined the ability of flagellin to interact with and stimulate the AP surface of IEC. Using various well-established IEC grown on transwell filters to develop a polarized monolayer with well-defined tight junctions, we exposed the AP surface of healthy human IEC cultures to purified flagellin from pathogenic and commensal bacteria or ND1/2ECHCD2/1 flagellin. We found that flagellin did not disrupt the integrity of the intestinal barrier, but did induce innate immune responses, such as IL-8 secretion and immune cell migration through IEC. Subsequently, the monomeric flagellins were internalized by IEC through a TLR5-dependent process. Once internalized, flagellin co-localized with endosomal surface markers at early time points and lysosomal markers at later time points.

## Materials and Methods

### Intestinal cell lines and cell cultures

Human colon adenocarcinoma intestinal epithelial cells, Caco-2BBe (American Type Culture Collection, Rockville, MD). were grown in Dulbecco’s modified Eagle’s medium (DMEM) supplemented with 10% fetal bovine serum (FBS), 110 mg/mL sodium pyruvate, and antibiotics. Human intestinal T-84 cells was grown in 50% DMEM and 50% Ham’s F-12 medium supplemented with 10% FBS and antibiotics. For experiments, Caco-2BBe and T-84 cells were grown on 0.4- or 3.0- µm transwell filters (Corning Inc., Corning, NY) depending upon the study design. IEC-18 cells (ATCC), a non-transformed rat intestinal epithelial cell line derived from undifferentiated crypt epithelial cells, were cultured with ATCC complete growth medium (Dulbecco's modified Eagle's medium with 4 mM L-glutamine adjusted to contain 1.5 g/L sodium bicarbonate and 4.5 g/L glucose) supplemented with human insulin solution at 1 mL per liter of medium (Sigma) and 5% fetal bovine serum (FBS). For experiments, IEC-18 cells were grown on 35 mm chamber dishes (MatTek). Confluence was verified by transepithelial electrical resistance (TER) of at least l000Ωcm^2^. Prior to experiments, the cells were washed once with appropriate cell medium containing no FBS then re-fed with the same serum-free medium. All experiments were performed using serum-free culture medium to ensure no potential interference with the flagellin protein interaction with the host cell.

### psiRNA TLR5 knock down

Knock down of specific TLR expression was accomplished by transfection of short-hairpin RNA plasmid-based constructs that targeted TLR5 (psiRNA system, InVivogen, San Diego, CA). Transfection of 80% confluent Caco-2BBe monolayers in 100 mm^2^ culture dishes was accomplished with FuGene (Roche, Indianapolis, IN) followed 48 h later by selection of stable transfectants with 250 ug/ml of zeomycin (Invitrogen, Carlsbad, CA). Specific target knock down was 70–80% based upon RT-PCR analyses.

### Detection of TLR5 on IEC

TLR5 expression was determined in the TLR5 knock down cells via Western blot analysis [13], [20] as compared to wild type Caco-2BBe cells. Further, wild type Caco-2BBe cells were infected with *S. typhimurium* (MOI 500) for 2 h then total cell protein was then collected from all samples followed by Western blot analysis [13], [20]. TLR5 was detected by using a primary rabbit polyclonal anti-TLR5 antibody (H-127; Santa Cruz Biotechnology) at the manufacturers recommended dilution (1∶1000).

### Flagellin proteins

Purification of native flagellin from *S. Typhimurium* [21] was performed as previously described. Recombinant 6-histidine (6HIS)-tagged flagellin protein from *S. dublin*, *E. coli* O83:H1 (organism was generously provided by Dr. Alexander Swidsinski, Charite Humboldt Universitat, Berlin, Germany), *E. coli* K12 and CBir1 were generated as previously described [13], [22], [23].

To generate fluorescently flagellin, *Salmonella dublin* was grown to late log phase in LB and then prepared for electroporation by incubation for 1 h at 50°C before centrifugation. Bacterial pellets then were washed 3X in 10% glycerol and were electroporated (Gene Pulser, BioRad) at 25uF, 1.8 kV, 400 (Pulse controller) with a pPROEX plasmid DNA encoding *S. dublin* flagellin tagged with GFP cloned into frame within the hypervariable region. The GFP was codon-optimized for bacterial expression and produced a chimeric flagellin with capacity to activate TLR5 that was easily visualized by fluorescent microscopy. Following electroporation, individual GFP positive colonies were selected by UV illumination. Log phase cultures of *S. dublin* expressing GFP-flagellin were treated with 10 mM IPTG to induce protein production prior to use. The identity of GFP-flagellin protein was confirmed by immunoblot analysis using mouse monoclonal anti-GFP (Roche).

### LPS detection in purified flagellin preparations

As we previously reported [13], [20] the purified flagellin was passed over an endotoxin removing gel column (Pierce) and found that there was <20 pg of LPS contaminated the purified flagellin as measured by the chomogenic Limulus ameboctye assay. Our previous findings determined that <1 µg/mL of LPS is unable to stimulate proinflammatory responses in IEC [13], [20]. To further eliminate the possibility of LPS interference, 10 µg/mL polymyxin B (PB), an LPS scavenger, was added to the purified flagellin 30 min prior to its addition to cell cultures.

### Cell fractionation

Caco-2BBe cells were seeded onto 0.4-µm transwell filters until confluent (approximately 14d). Monolayers were rinsed with ice-cold DMEM containing 20 mM Hepes, pH 7.4, 0.1% BSA. To specifically label endocytic traffic up to and including recycling endosomes cells [24], [25] seeded transwell cultures were incubated at 19°C for 30 min with flagellin (0.4 mg/mL) added either to the basolateral (BL) or apical chamber. Biotinylated-Transferrin (50 mg/mL Bt-Tfn; Molecular Probes) and flagellin was simultaneously added to the AP chamber. Cultures then were immediately analyzed (without chase to avoid further endosomal maturation). To evaluate subsequent protein traffic, following the 19°C incubation cells were rinsed with ice-cold medium and then chased for 30 min or 2 h in pre-warmed (37°C) medium.

To isolate cellular fractions subsequent manipulations were carried out at 4°C. Organelles were fractionated on a 1–16% ficoll gradient as previously described [26]. Briefly, cells were rinsed with 0.32 M sucrose, 10 mM Hepes, pH 7.4 (HB), then scraped into 1 ml HB, and homogenized by nine passages through a ball-bearing homogenizer (0.012-mm clearance; EMBL, Heidelberg, Germany). The homogenate was spun at 11,000 × g for 5 min, and 1 ml of this post-nuclear supernatant was layered on 11 ml of a 1–16% linear ficoll gradient in HB. The gradients were centrifuged for 45 min at 35,000 rpm in a SW40Ti rotor (Beckman Instruments, Palo Alto, CA) and fractions collected from the top of the tube using an Autodensi-Flow I1C (Buchler Instruments, Kansas City, MO).

To further fractionate early endosomes and the late endosomal/lysosomal compartments, fractions were subjected to 2 different gradients as previously described. Tfn positive early endosomes were run on a 9 ml 3–16% 2nd gradient in a SW40Ti rotor for 50 min at 35,000 rpm as previously described [27]. Late endosomal/lysosomal (beta-glucuronidase positive) fractions were pooled and run on a 9 ml 7–25% gradient in SW40Ti rotor for 45 min at 35,000 rpm [28], [29]. Fractions were collected as above and subjected to 5–15% SDS PAGE, transferred to PVDF, and probed with anti-His (for flagellin), anti-Cathepsin D (mature form; Sigma), or Streptavidin-peroxidase (SA-HRP; to recognize Bt-Tfn, Sigma). Antibodies were detected with peroxidase-coupled secondary antibodies. The presence of mature Cathepsin D, Bt-Tfn (SA-HRP), and flagellin (His) was quantified from films using densitometry with a ChemImager 4400 and AlphaEase software (Alpha-Innotech; San Leandro, CA). The value obtained from each fraction was normalized by division with the value obtained from the post-nuclear supernatant, run in parallel.

### Determination of flagellin internalization *in vitro* with confocal and epifluorescence microscope

Flagellin (5 µg) or 1×109 fluorescein-conjugated dextran beads (molecular weight of 40,000; Molecular Probes) were added to the apical surface of polarized IEC. Caco-2BBe and T-84 filters were fixed at selected time points with 2% formaldehyde for 5 min and then permeabilized with 0.2% Triton X100 for 7 minutes. Internalized flagellin was detected with rabbit anti-flagellin (Protein Express, Cincinnati, OH) followed by labeling with an anti-rabbit secondary antibody conjugated to the indicated fluorescent dye (Kirkegaard and Perry Laboratories, Gaithersburg, MD). Additional filters exposed to flagellin were stained with 2 µg/mL of rabbit anti-ZO-1 antibody and fluorescently labeled secondary antibody (Zymed, San Francisco, CA) for 1 hour to examine polarized cell tight junctions. After PBS rinsing, filters were blocked for 30 min with 10% nonfat dried milk resuspended in Tris-buffered saline (TBS) prior to incubation with a goat polyclonal anti-EEA1 antibody (Santa Cruz Biotechnology, Inc.) to stain early endosomes or a mouse monoclonal anti-LAMP-1 antibody (Stressgen, Ann Arbor, MI) to stain lysosomes, for 1 h at a 1∶10,000 dilution. Filters were washed extensively and then incubated with the appropriate secondary antibody conjugated to FITC or rhodamine (Kirkegaard and Perry Laboratories) diluted 1∶10,000 for 1 h. Phalloidin (Alexa Fluor 633, Molecular Probes) was used to visualize F-actin. After final washes, the filters were cut from the transwell and mounted cell side up on a microscope slide. Mounting solution containing 4’, 6-diamidino-2-phenylindole (DAPI) to label the cell nucleus (*Slow*Fade® Antifade kit, Molecular Probes, Eugene, OR) was used to mount a cover slip on top of the filter.

To determine flagellin internalization and localization of CBir1 flagellin, the chamber slides with confluent cell monolayers were washed with cold PBS, added fresh pre-cold culture medium, and set on ice for 10 min. Then, dual-labeled flagellin “Alex488+Biotin-Flax” (15 µg/ml) was added to medium. Chamber slides were set on ice for additional 30 min to allow the flagellin adhering on the cell surface. Thereafter, cells were cultured in a water-saturated atmosphere with 5% CO_2_ at 37°C for 2 h. At the end of incubation, cells were washed three times with ice-cold PBS, fixed with 4% paraformaldehyde (pH 7.2) for 10 min, and washed with PBS three times. To label extracellular biotinylated flagellin, fixed-cells were incubated with strepavidin-Alex 633 (1∶500 dilution, invitrogen) for 30 min at room temperature followed by washing with PBS three times. The nuclei were counterstained with DAPI. Finally, slides were examined with a fluorescence microscope (MDR, Leica). The internalized or external flagellin particles were identified from Alexa Fluro 488 fluorescence, whereas only extracellular flagellin particles were visualized by the Alexa Fluor 633 [19]. The time of measurements, image capturing, and image intensity gain at all wavelengths were optimally adjusted and kept constant. Images were analyzed by image-analysis software (Openlab1) and assembled with Adobe Photoshop 8.01 software.

Confocal images of fixed IEC were acquired using a Zeiss LSM510 confocal microscope system with 60x objective lens and Axiovision software package (Carl Zeiss Inc, Germany). For color images, multitract confocal acquisition [using separate lasers (argon, 488 nm; HeNe, 543 nm) for excitation] was used in acquiring confocal images. Filter sets were optimized to eliminate fluorescence overlap and bleed through. In order to examine intracellular flagellin, confocal images of polarized IEC grown on transwell filters were optical z-section images, at the indicated distance apart, were obtained and compiled into a z-stack representing the cell monolayer in all 3 dimensions.

### Blocking of apically expressed TLR5

Caco-2BBe cells were grown on transwell filters as previously described above. TLR5 blocking peptide (H-18; Santa Cruz Biotechnology) was added, at a dilution (1∶100) recommended by the manufacturer, to the AP surface of Caco-2BBe cells and incubated at 5%CO_2_/37°C for 30 min, followed by the addition of 5 µg of purified recombinant 6HIS-tagged *S. dublin* flagellin for 1 h. Caco-2BBe cells incubated with flagellin alone served as controls. The AP and BL supernatants were collected as well as total cell protein from Caco-2BBe cells [20]. Supernatants and cell protein was analyzed via Western blot as previously described [13], [20]. Blots were immunolabelled using a mouse anti-6HIS antibody (1: 2000; Roche) to detect recombinant flagellin monomers.

### Human DC and PMN

DCs were isolated from monocytes purified from PBMC by negative selection using the magnetic column separation system (StemCell Technologies, Inc.) as previously described [30]. Briefly, purified monocytes were cultured in RPMI 1640 medium supplemented with 10% FCS, 5% L-glutamine, Hepes, 5% sodium pyruvate, 5% antibiotics, GM-CSF (100 ng/mL) and IL-4 (50 ng/mL) and set up in 24-well tissue culture plates at 5×10^5^/mL. Non-adherent, immature DCs were obtained at 7 days of culture and only homogeneous immature DC populations, characterized by high levels of CD11a and no CD83 expression, were used in these experiments.

PMN purification was performed as previously described [31]. Briefly, anti-coagulated venous blood was collected from healthy volunteers following written consent under an approved protocol and consent procedure (#07-177) by the University of Texas Medical Branch Institutional Review Board (IRB). PMN were isolated using dextran sedimentation and Hypaque-Ficoll (Amersham Biosciences, Piscataway, NJ) density-gradient separation. Purified PMN were resuspended in RPMI 1640 before use in migration experiments.

Caco-2BBe or T-84 cells, grown on inverted 3- µm filter then 100 ng/mL of *S. dublin*, ECO83:H1 or *E. coli* K12 full-length flagellin, ND1/2ECHCD2/1 or D3 flagellin construct was added to the BL chamber to stimulate the AP surface of IEC. Flagellin dose(s) and time points in these studies were chosen as a result of our previous work [20], [25]. After 2 h of pre-exposure to one of the flagellins, freshly isolated DC or PMN (1×10^5^) were added to the AP chamber and migration assessed at 4 h via cell counts. DC or PMN on un-stimulated polarized cells served as un-stimulated controls. The AP and BL supernatants were collected for counting in a hemocytometer to determine immune cell migration. Additionally, AP and BL supernatants from DC and PMN migration were analyzed to evaluate cytokine secretion.

### Detection of IL-8

Transwell filter Caco-2BBe or T-84 supernatants were collected at 6 h following flagellin stimulation. The time point to measure IL-8 secretion was based on our previous experience examining this cytokine in IEC [13], [20]. IL-8 levels in the supernatants were quantitated by a human IL-8 ELISA kit (Pierce/Endogen).

### Statistical analysis

Experimental conditions were performed in duplicate, and experiments were repeated at least twice to ensure reproducibility. Statistical differences were considered significant if the p value was 0.05 as determined by ANOVA followed by Student’s t-test. Where appropriate, data are expressed as mean ± standard deviation of the mean (SD).

## Results

### Internalization of flagellin by polarized IEC

**Figure 1:**
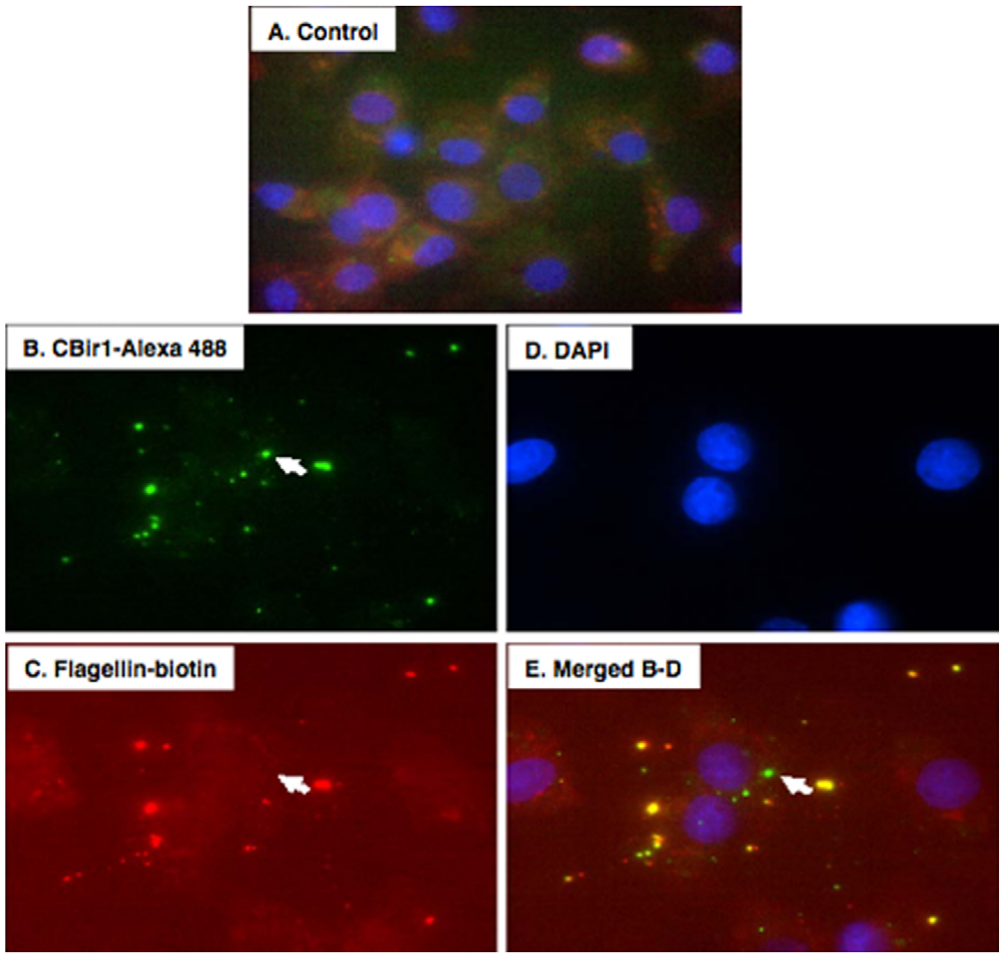
Flagellin is taken up by rat IEC monolayers. **A.** Control IEC-18 cells that were not exposed to CBir1 flagellin. **B and C.** Following a 2 h incubation, CBir1 was internalized by IEC-18 cells as detected flagellin labeled with Alexa-488 (green) and biotin. **E** represents merged images of **B-D** showing the dual stained flagellin as visualized by fluorescent microscopy. Cell nuclei were stained with DAPI (blue).

In a pilot experiment, we examined whether flagellin was taken up by IEC. To this end, confluent, non-polarized IEC-18 cells were incubated with dual-labeled CBir1 flagellin. To detect the internalized flagellin, we used techniques described in our previous work [19]. Results demonstrated that flagellin was found inside IEC-18 cells 2 h post-flagellin exposure (Fig. 1A–E), revealing that IEC are able to engulf flagellin in vitro.

**Figure 2:**
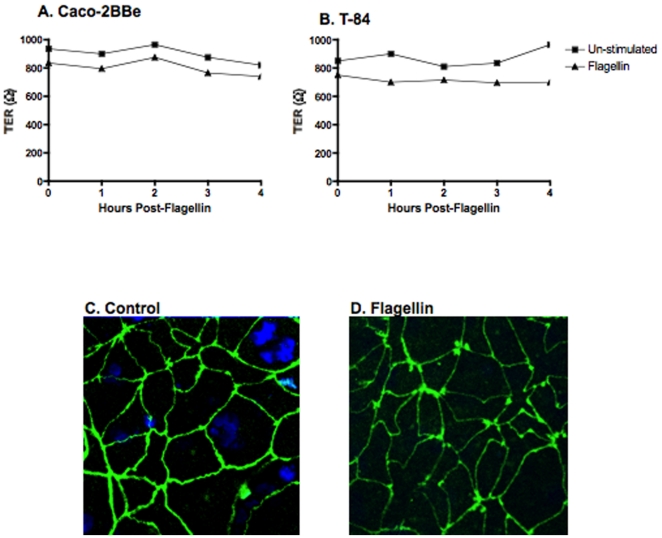
Flagellin does not interfere with intestinal barrier integrity. TER measurements were taken every hour for 4 h in the AP and BL chambers of polarized Caco-2BBe (**A**) and T-84 (**B**) cells exposed to flagellin that showed no significant change. **C** and **D**. Examination the tight junction protein, ZO-1 by confocal microscopy, in polarized Caco-2BBe cells. The appearance of the junctions stained with anti-ZO-1 antibody and appropriate secondary antibody (green) did not change following exposure of flagellin to the AP surface. Cell nuclei with stained with DAPI (blue). The confocal images shown are representative of 2 experiments.

To study whether internalization of flagellin is through a trans- or para-cellular pathway, we first examined whether the AP exposure of human IEC to flagellin would affect the barrier integrity of the monolayer. As such Caco-2BBe and T-84 cells were grown on transwell filters and TER in the AP and BL chambers was measured simultaneously every hour for 4 h following the addition of purified *S. dublin* flagellin to the AP surface. Results showed that the TER of Caco-2BBe (Fig. 2A) and T-84 (Fig. 2B) did not change significantly at any of the time points measured following incubation with flagellin compared to un-stimulated cells (Fig. 2A and B). Further, the tight junctions remained intact and unaffected following AP flagellin exposure as compared to un-stimulated controls (Fig. 2C and D) demonstrating that flagellin does not compromise the integrity of the IEC barrier and it remains preserved suggesting that flagellin is traveling a trans-cellular route.

**Figure 3:**
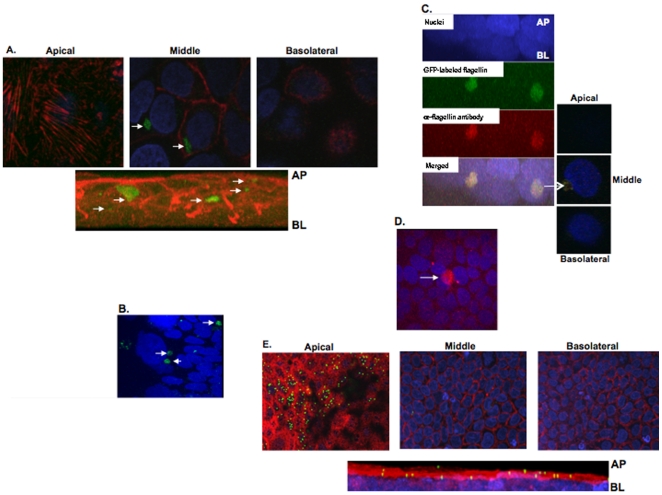
Flagellin monomers were internalized by polarized IEC. Note: White arrows in A-D indicate cluster of flagellin monomers. **A.** GFP-labeled flagellin monomers (green) were internalized by Caco-2BBe cells at 30 min post-AP exposure and visualized via confocal microscopy. The cell body was visualized by staining for F-actin using phalloidin (red). Accumulated flagellin was detected inside the cells as shown in the mid z-sections taken by confocal microscopy. A transverse image reconstruction from optical z-stacks is shown below and shows flagellin below the cell surface. **B**. GFP-labeled flagellin was added to the AP surface of polarized T-84 cells and 30 min post-exposure flagellin clusters could be detected within the cell body from mid z-sections taken by confocal microscopy using a 60x objective lens. Cell nuclei were stained by DAPI (blue). **C**. Caco-2 BBe cells internalized GFP-labeled flagellin (green) which was also immunostained using a rhodamine rabbit anti-flagellin antibody (red). Co-localization GFP and rhodamine signals is seen in the merged images and in the transverse image reconstruction from z-stacks taken by confocal microscopy (at right). **D**. Flagellin purified from *S. Typhimurium* was internalized by Caco-2BBe cells. Intracellular staining of purified *S. Typhimurium* flagellin were visualized, via the middle z-section taken by confocal microscopy after 30 min of apically applied flagellin using a rabbit anti-flagellin antibody followed by a secondary anti-rabbit antibody conjugated to rhodamine (red). Cell nuclei were stained with DAPI (blue) **E**. Optical z-sections showing apical and basolateral surfaces as well as the middle plane through the cells together with a transverse image reconstructed from z-stacks showing lack of uptake of latex beads (MW 40,000; green) by Caco-2BBe cells after a 30 min incubation. F-actin was stained using phalloidin (red) and cell nuclei were stained with DAPI (blue). Images were acquired using a 60X objective lens. The confocal images shown are representative of 1 of 4 experiments producing similar results.

We then examined whether IEC were capable of internalizing apically applied flagellin. Therefore, 5 µg of GFP-labeled *S. dublin* flagellin protein was added to the AP surface of polarized Caco-2BBe cells. Latex beads (molecular weight 40,000) were added to the AP surface of a parallel set of Caco-2BBe cultures as a non-specific uptake control. Following a 30 min incubation period, cells were washed vigorously then fixed and immunolabeled to visualize specific organelles. The middle X-Z cell slices revealed that GFP-labeled flagellin were internalized by Caco-2BBe cells (Fig. 3A) with similar outcomes generated in polarized T-84 cells (Fig. 3B). Confirmation that the green fluorescence was in fact flagellin was obtained by immunolabeling with a rabbit anti-flagellin polyclonal antibody and a secondary goat anti-rabbit antibody conjugated to rhodamine (merged X-Z reconstruction images; Fig. 3C). Completion of the same study with purified, native flagellin isolated from *S. Typhimurium* produced the same intracellular localization in AP-treated Caco-2BBe cells (Fig. 3D) as GFP-flagellin (Fig. 3A). Caco-2BBe cells did not internalize the latex beads that remained dispersed on the apical surface of the cells (Fig. 3E).

### TLR5-mediated internalization of flagellin

**Figure 4:**
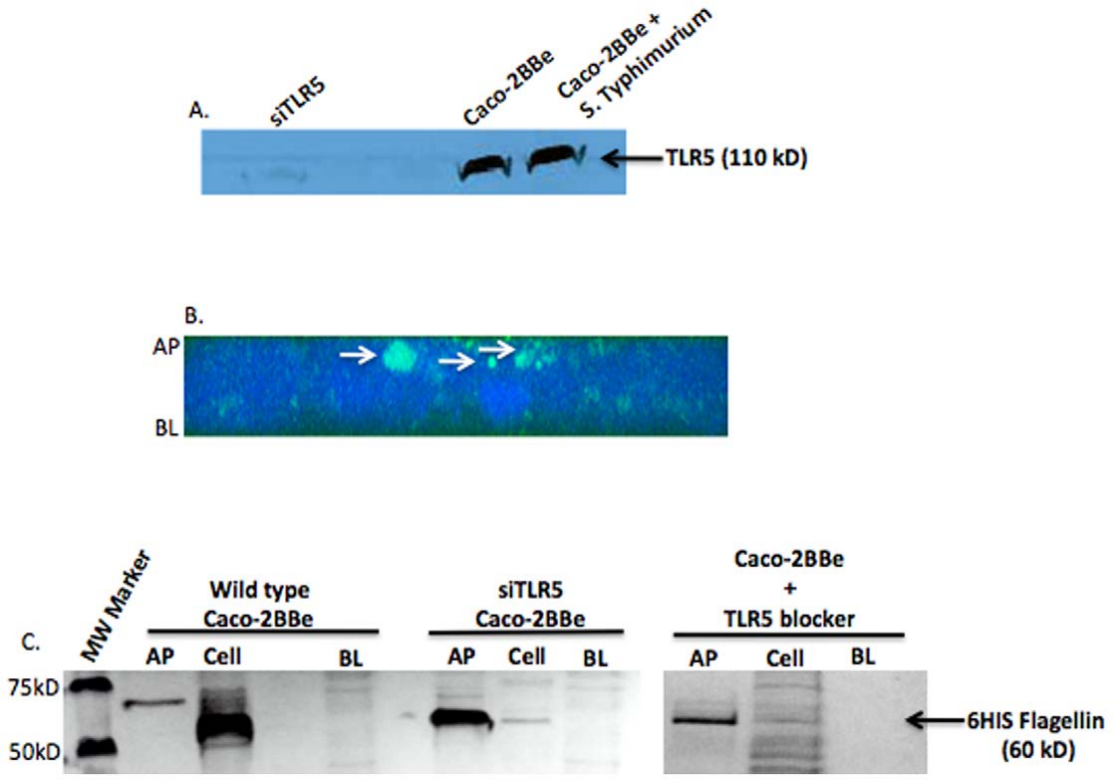
Flagellin was not internalized by siTLR5 Caco-2BBe cells or IEC in which TLR5 was blocked. **A**. Immunoblotting for TLR5 via Western blot showed that TLR5 expression is dramatically reduced in siTLR5 Caco-2BBe cells compared to wild type Caco-2BBe cells. Further, wild type Caco-2BBe cells infected with *S. Typhimurium* (MOI 500) did not change the expression of TLR5 2 h post-infection. **B.** Transverse image of polarized siTLR5 Caco-2BBe cells constructed from optical z-stacks taken by confocal microscopy, showing flagellin (green) was localized to the AP surface at thirty minutes post-exposure. Cell nuclei were stained with DAPI (blue). **C**. The majority of flagellin remained on the apical surface of siTLR5 Caco-2BBe cells and wild type Caco-2BBe cells pre-treated with TLR5 blocker antibody following 1 h incubation with 5 µg of flagellin. Whereas, the predominant quantity of flagellin was found in the cell layer of wild type Caco-2BBe controls. Flagellin was not detected in the BL supernatants of any of the tested cell samples. These results represent 1 of 3 repeated experiments with similar results.

To determine if TLR5 played a role in mediating the internalization of flagellin, TLR5 siRNA encoding plasmid was used. Caco-2BBe cells were selected that had stably reduced TLR5 expression (siTLR5 cells). Western blot analysis confirmed that TLR5 expression was suppressed as compared to wild type Caco-2BBe cells (Fig. 4A). The siTLR5 cells were grown on transwell filters to confluence prior to the addition of GFP-flagellin to the AP surface as described above. Following 30 min incubation, flagellin was not visible inside the cells by confocal microscopy. Instead, the flagellin (green) remained at the apical surface of the cells (Fig. 4B).

To further confirm the role of TLR5 in the internalization of flagellin, purified recombinant flagellin was added to the apical surface of siTLR5 Caco-2BBe cells or wild type Caco-2BBe cells pre-treated with a TLR5 peptide that blocked binding of flagellin to the TLR5 receptor. Following a 1 h incubation with flagellin, results showed that the majority of flagellin remained in the AP supernatant of siTLR5 Caco-2BBe cells and Caco-2BBe cells pre-treated with a TLR5 blocker compared to wild type Caco-2BBe cells where the majority of flagellin was detected in the cell layer (Fig. 4C). Flagellin was not detected in the basolateral (BL) chamber of siTLR5 Caco-2BBe cells, Caco-2BBe cells treated with TLR5 blocker peptide or wild type Caco-2BBe cells (Fig. 4C).

Thus far we have shown that purified flagellin is internalized by IECs via TLR5 but does the presence of the live intestinal pathogen, *S. Typhimurium*, affect the expression of TLR5 on IECs perhaps down regulating the receptor subsequently reducing the binding of luminal flagellin. To test this hypothesis, wild type Caco-2BBe cells, grown on transwell filters, were infected for 2 h with *S. Typhimurium* (MOI 500). Results showed that *S. Typhimurium* did not affect TLR5 protein expression, either up- or down-regulation of the receptor, which was similar to uninfected cells as analyzed by Western blot (Fig. 4A).

The culmination of these data demonstrates that flagellin is internalized from the AP surface of various IEC lines, which is mediated by TLR5. Further, the lack of flagellin detection in the BL supernatant of wild type Caco-2BBe cells is an indication that flagellin is maybe degraded following IEC internalization.

### Intracellular flagellin co-localized with endosomal and lysosomal associated proteins

**Figure 5:**
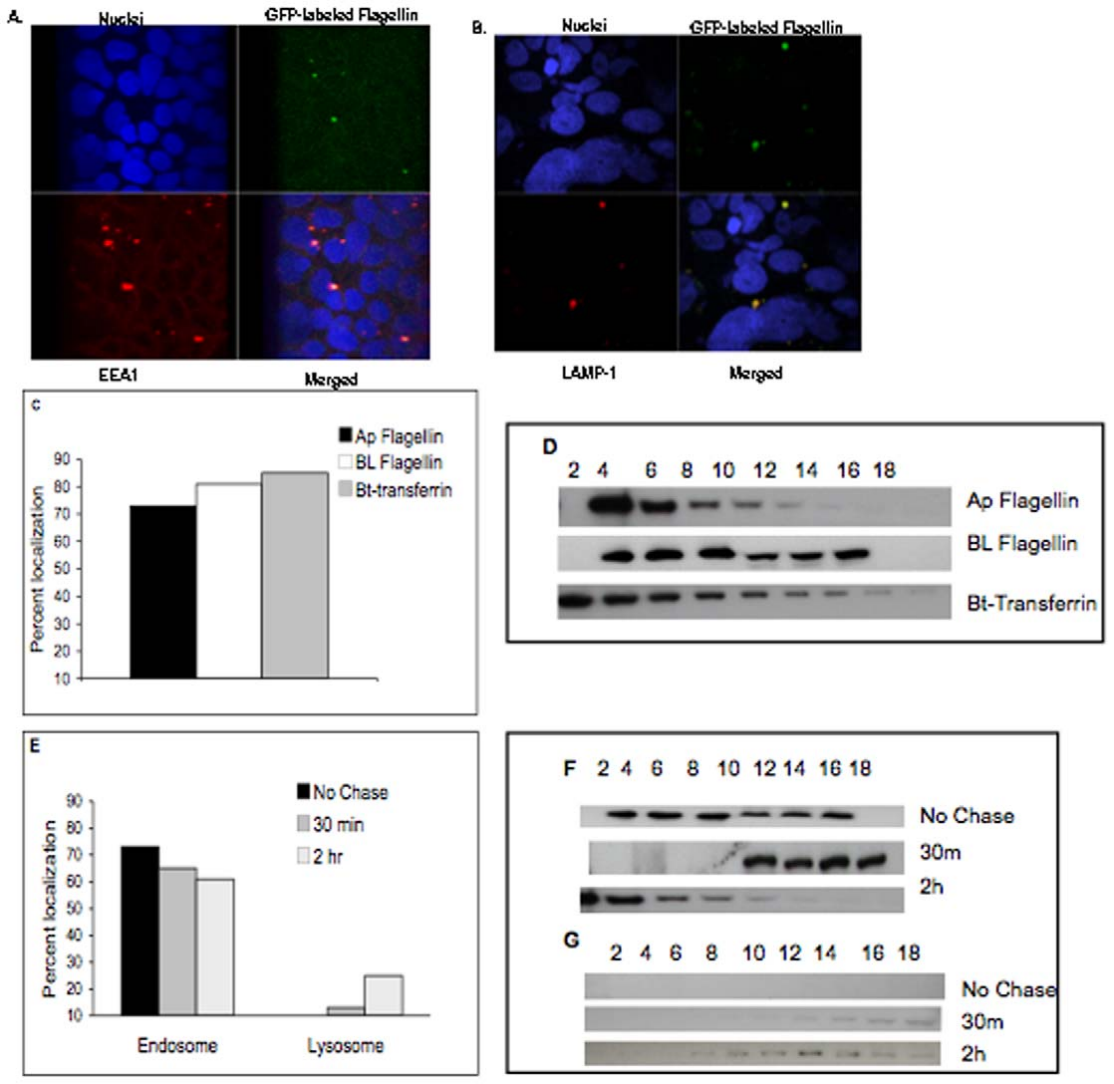
Flagellin co-localizes with endosomal and lysosomal markers. **A**. Following AP exposure to Caco-2BBe cells, GFP-labeled flagellin (green) was internalized and, within 15 min, co-localized with an antibody that recognized an early endosomal surface marker (EEA1) and secondary antibody (red) as shown in the middle z-sections by confocal microscopy. Lower right panel shows merged images and indicates co-localization of flagellin with EEA1.Cell nuclei were stained with DAPI (blue). **B**. Within 1 h flagellin (green) co-localized with the lysosome marker LAMP-1 (red). The confocal images shown are representative of 3 independent experiments. **C**–**F**. 6HIS-Flagellin was added to the AP or BL side of polarized Caco-2BBE cells at 19°C allowing traffic up to (but not beyond) the recycling endosome. In some experiments biotinylated transferrin also was added to the apical surface as a marker for the endosomal pathway. Cells then were either fractionated (see methods) without allowing further traffic or warmed to 37°C for the times indicated prior to fractionation. NOTE: White arrows indicate flagellin protein in A and B. **C**. Intracellular localization of internalized flagellin protein (normalized for total cell-associated protein) following incubation at 19°C. **D**. Western blots of endosomal fractionation as described in C and in the methods. **E**. Intracellular localization of 6HIS-Flagellin when internalization of protein at 19°C was followed by incubation at 37°C allowing traffic beyond the recycling endosome. **F-G**. Western blots of fractionation as described in E and in the methods.

To identify the intracellular compartment containing internalized flagellin, the AP surface of polarized Caco-2BBe cells were exposed to GFP-flagellin for 15 or 60 min. Cells then were washed thoroughly, fixed, permeabilized, and processed for immunofluorescence using antibodies directed against EEA1, an early endosome-specific protein that co-localizes with the transferrin receptor [32] or LAMP-1, a lysosome associated membrane protein. Immunofluorescence detection of EEA1 revealed co-localization with GFP-flagellin at 15 min post-exposure to flagellin (Fig. 5A). LAMP-1 was found to co-localize with GFP-flagellin at the later (60 min) time point (Fig. 5B) consistent with sequential trafficking through these endocytic compartments. Figures 5A and B show middle (intracellular) z-sections of cells from confocal microscopy.

To further elucidate the co-localization of flagellin within cellular vesicles, cell fractionation was performed on polarized Caco-2BBe cells incubated with flagellin. Confocal microscopy indicated that the 6-HIS flagellin protein traffics indistinguishably from native or GFP-tagged flagellin purified directly from *S. Typhimurium*. Because larger quantities of the 6-HIS flagellin protein were easier to obtain, this recombinant flagellin was utilized to quantify the intracellular trafficking by sub-cellular fractionation followed by immunoblot. Incubation at 19°C has been shown to allow internalization from both the AP and BL surfaces and delivery to the shared recycling endosome but to inhibit further trafficking [24], [25]. We, therefore, added flagellin to the AP or BL surface of polarized Caco-2BBe cells at 19°C and followed intracellular trafficking as described in the methods. When cells were fractionated without warming (trafficking inhibited beyond the recycling endosome), 73% of the flagellin added to the AP surface co-localized with the clathrin-mediated endocytic marker, transferrin (Fig. 5C). When the flagellin was added to the BL surface 81% co-localized with transferrin (Fig. 5C; this difference was within experimental error). Under these conditions there was no co-localization with the lysosomal (mature) marker, cathepsin D. Trafficking subsequent to the recycling endosome should be the same regardless of the surface that internalized it. Figure 5D presents Western blots of endosomal fractionation representative of the data shown in figure 5C.

In a second set of experiments, excess flagellin was removed from the medium and then the cells were warmed to 37°C for 30 min in fresh medium. Following this process, 65% of the flagellin still co-localized with transferrin but 13% co-localized with mature cathepsin D indicating that flagellin had moved into lysosomes (Fig. 5E–F; F–G are Western blots of fractionation as shown in E). When the 37°C incubation was extended to 2 h, the amount of flagellin that co-localized with transferrin was 61% while 25% of the flagellin co-localized with mature cathepsin D in lysosomes (Fig. 5E). The half-life of GFP-tagged flagellin was not compared to the wild type flagellin protein purified from *S. Typhimurium* however, the amount of flagellin present was within the range of deviation seen with transferrin (that recycles to the cell surface) indicating that the chimeric flagellin, like wild type, was degraded slowly in lysosomes (data not shown).

### Stimulation of polarized IEC with flagellin induced an inflammatory response and immune cell migration

**Figure 6:**
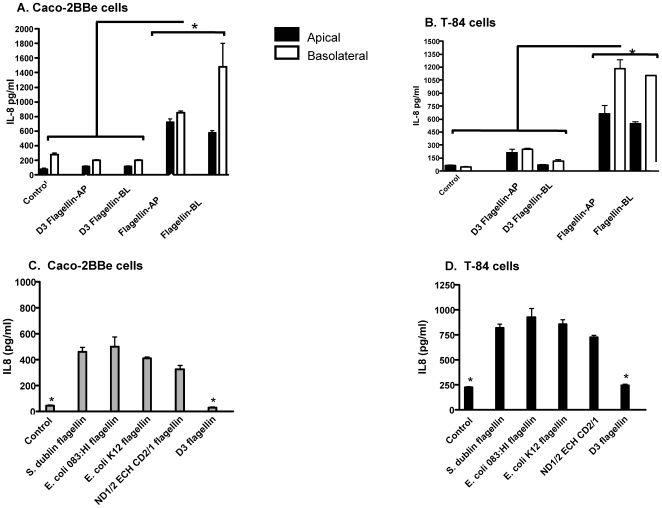
IEC AP or BL exposure to flagellin induces IL-8 secretion. Purified Salmonella flagellin or D3 recombinant flagellin was added to the AP or BL chamber of Caco-2BBe (**A**) or T-84 (**B**) cells grown on transwell filters. Supernatants were collected at 6 h post-exposure and analyzed for IL-8 secretion. Both cell lines (**A** and **B**) secreted IL-8 regardless of AP or BL stimulation with full-length flagellin compared to the D3 protein and un-stimulated cells. However, higher levels of IL-8 were detected in the BL supernatant compared to the AP supernatant. Further, Caco-2BBe (**C**) and T-84 (**D**) cells were apically stimulated with various types of flagellin proteins. Un-stimulated cells served as negative controls. *S. dublin* flagellin, ECO83:HI flagellin, *E. coli* K12, ND1/2 ECH CD2/1 flagellin induced higher IL-8 secretion from the BL surface of both cells types compared to D3 truncated flagellin or un-stimulated controls. Data is expressed as mean ± SD. *p<0.05 vs. all groups.

To determine if the AP as well as the BL surface of two well-established IEC was capable of responding to various flagellin proteins, 100 ng/mL of purified flagellin from *S*. *dublin* or D3 was added to the AP or BL surface of polarized Caco-2BBe or T-84 transwell cultures and IL-8 was quantitated in the cells supernatants. All experiments were performed in the presence of 10 µg/mL of polymyxin B to ensure the measured inflammatory responses were flagellin-induced rather than LPS-mediated. Our results showed the addition of flagellin to the AP or BL surface of IEC induced IL-8 secretion from both surfaces (Fig. 6A and B). Specifically, significant IL-8 levels were detected in the AP and BL supernatant of Caco-2BBe and T-84 cells following addition of *S. dublin* flagellin to the AP chamber compared to AP supernatants from D3 and untreated controls (Fig. 6A; P<0.05). Further, the secretion of IL-8 from the BL surface was higher compared to IL-8 secretion from the AP surface regardless of whether flagellin was added to the AP or BL chamber (Fig. 6A and B).

These data demonstrate the responsiveness of the AP surface to flagellin from *Salmonella* via IL-8 secretion so we wanted to determine if the AP addition of various types of flagellin from virulent, avirulent, and truncated flagellin monomers would induce the same or a similar reaction from IEC. Therefore, as described above, 100 ng/mL of purified flagellin from *S*. *dublin*, E. coli 083:H1, or *E. coli* K12 or truncated *S. dublin* ND1/2ECHCD2/1 or D3 flagellin were added to the AP surface of polarized Caco-2BBe or T-84 cultures. Results showed that flagellin from both virulent and avirulent bacterial strains applied to the AP surface of Caco-2BBe (Fig. 6C) or T-84 (Fig. 6D) cells induced comparable levels of IL-8 secretion from the BL surface within 6 h post-stimulation. IL-8 secretion was higher overall in flagellin-stimulated T-84 cells (Fig. 6D) compared to flagellin-stimulated Caco-2BBe cells (Fig. 6C) but the difference was not significant. Full-length flagellin proteins, from *S. dublin*, *E. coli* 083:H1 and *E. coli* K12, and truncated flagellin containing only the conserved N and C domains (ND1/2 ECH CD2/1) stimulated nearly equivalent levels of IL-8 secretion from both cell lines (Fig. 6C and D). As observed in Figure 6, the D3 flagellin protein did not trigger IL-8 secretion from IEC cultures compared to all other tested flagellins (Fig. 6C and D; p<0.05).

**Figure 7:**
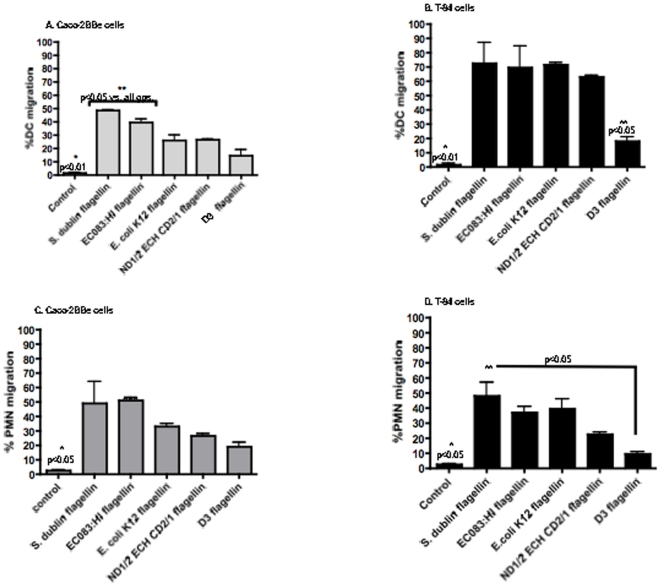
Immune cell migration through IEC in response to luminal exposure to flagellin. Polarized Caco-2BBe and T-84 cells were stimulated with flagellin proteins 2 h prior to the addition of human DCs (**A** and **B**) or neutrophils (**C** and **D**) to the BL surface of IEC. Supernatants were collected 4 h post-migration and immune cells were counted to determine migration from the BL to the AP surface of IEC in response to flagellins. Data is expressed as mean ± SD. In Figure 2A, *p<0.01 vs. all groups except D3 flagellin and **p<0.05 vs. all groups. In Figure 2B, *p<0.01 vs. all groups except D3 flagellin and **p<0.05 vs. all groups excluding controls. Figure 2C, p<0.05 vs. *S. dublin* and ECO83:H1 flagellin. Figure 2D, *p<0.05 vs. all groups except D3 flagellin and **p<0.05 vs. D3 flagellin.

Because IL-8 is a classic chemoattractant for immune cell recruitment, we investigated transepithelial migration of DC and PMN added to flagellin-stimulated polarized Caco-2BBe and T-84 cells. To model the in vivo physiological situation of immune cell migration from the lamina propria to the intestinal lumen, flagellin from *S. dublin*, ECO83:H1, and *E. coli* K12 or recombinant ND1/2ECHCD2/1 or D3 proteins were added to AP surface of IEC, for 2 h prior to the addition of PMN or DC the BL surface of IEC. Immune cells added to un-stimulated cells served as negative controls. Following 4 h of migration, results showed that each of the flagellin proteins was capable of stimulating varying degrees of DC migration through both Caco-2BBe (Fig. 7A) and T-84 cells (Fig. 7B). Consistent with their ability to induce significant levels of IL-8, flagellin from *S. dublin* and ECO83:H1 induced significantly higher DC migration through Caco-2BBe compared to the other tested flagellin proteins or un-stimulated controls (p<0.05, Fig. 7A and B). *E. coli* K12 flagellin and ND1/2ECHCD2/1 induced significantly higher DC migration through Caco-2BBe cells compared to un-stimulated controls (p<0.05) but significantly less migration compared to *S. dublin* and ECO83:H1 flagellin proteins (p<0.01; Fig. 7A). However, migration through T-84 cells showed that flagellin proteins from *S. dublin*, ECO83:H1, *E. coli* K12, and ND1/2ECHCD2/1 induced equivalent levels of DC migration that was significantly higher than un-stimulated controls (p<0.01, Fig. 7B). As expected, the flagellin D3 region induced minimal DC migration in Caco-2BBe (Fig. 7A) and significantly lower migration in T-84 cells compared to the other tested flagellin proteins (p<0.05, Fig. 7B).

Similar migration trends were observed with PMN following apical addition of flagellin to polarized IEC cultures. Flagellin from *S. dublin* and ECO83:H1 stimulated significantly higher PMN migration through Caco-2BBe cells (Fig. 7C) than measured in un-stimulated controls (p>0.05). *E. coli* K12 flagellin and ND1/2ECHCD2/1 evoked PMN migration less than full-length flagellins but significantly more than the D3 flagellin protein (Fig. 7C). Similarly, maximal PMN migration was observed through T-84 cells stimulated by flagellin proteins from *S. dublin*, EC O83:H1 and *E. coli* K12 compared to un-stimulated cells (p<0.05; Fig. 7D). ND1/2ECHCD2/1 elicited less PMN migration through T-84 cells but again more than the D3 flagellin protein (p<0.05; Fig. 7D).

These results collectively indicate that flagellin proteins from virulent and avirulent bacterial strains as well as a flagellin truncated protein, containing the N and C conserved regions, stimulated the AP surface of IEC inducing IL-8 secretion and the subsequent migration of immune cells.

### Reduction of TLR5 via siRNA significantly decreased IL-8 secretion from flagellin-exposed IEC

**Figure 8:**
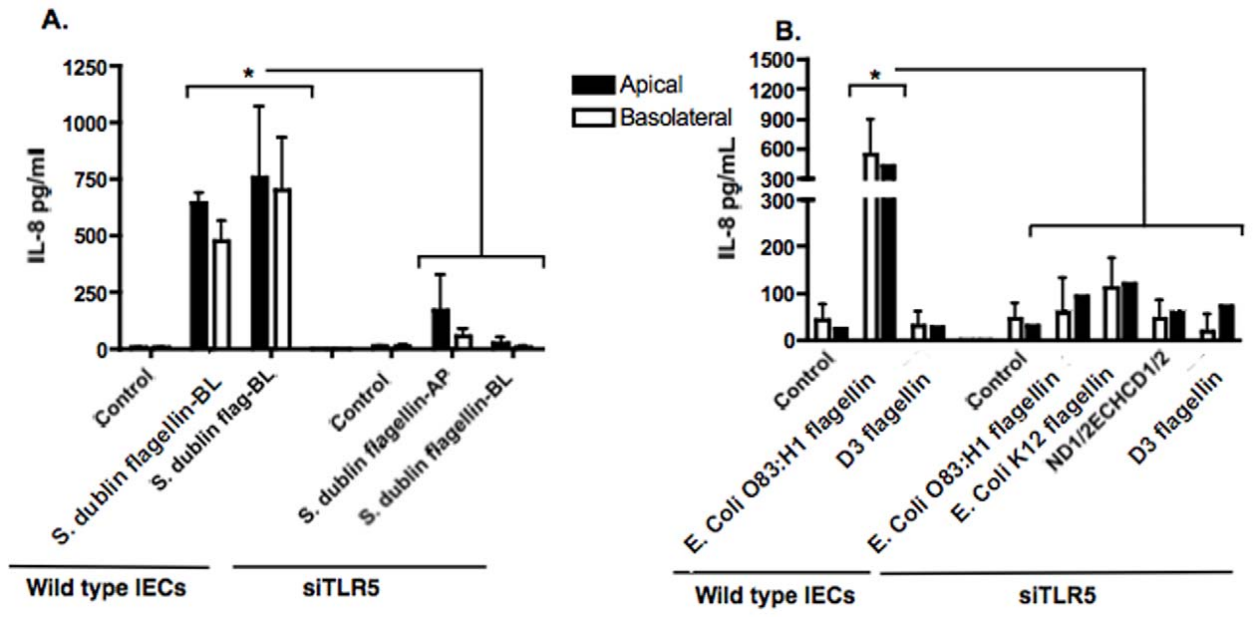
IL-8 secretion decreased from TLR5 silenced (siRNA) Caco-2BBe cells exposed to flagellin. **A.** *S. dublin* flagellin was added to the AP or BL surface of polarized Caco-2BBe cells where TLR5 was knocked down using siRNA. Parallel wild type Caco-2BBe cells were treated as above. Six hours post-flagellin exposure, AP and BL supernatants were collected to quantitate IL-8 secretion. IL-8 secretion was significantly decreased from siRNA Caco-2BBe cells stimulated with *S. dublin* flagellin regardless of AP or BL exposure compared to wild type Caco-2BBe cells. **B**. IL-8 secretion was significantly decreased in siTLR5 Caco-2BBe cells apically exposed to flagellin from *S. dublin*, ECO83, *E. coli* K12, and ND1/2ECHCD2/1 compared to wild type cells. Data is expressed as mean ± SD. *p<0.05 vs. all groups.

It has been reported that BL TLR5 sequestration is a biological process to distinguish commensal and pathogenic bacterial flagellin requiring that the protein cross the epithelial barrier to be recognized [33], [34]. However, we showed in figure 4 that intracellular flagellin was not detected in siTLR5 Caco-2BBe cells. Therefore, to expand on these findings, we wanted to determine if flagellin stimulates IL-8 secretion via TLR5 on the AP surface of IEC. Confluent siTLR5 Caco-2BBe cells were grown on transwell filters then the AP or BL surface of IEC were exposed to *S. dublin* flagellin as described previously. Regardless of whether flagellin was added to the AP or BL chamber, IL-8 secretion was significantly decreased in TLR5 reduced IEC exposed to *S. dublin* flagellin compared to the control IEC (p<0.05, Fig. 8A). Additionally, the AP exposure of siTLR5 Caco-2BBe cells to flagellin from ECO83, *E. coli* K12, or ND1/2ECHCD2/1 resulted in significantly decreased IL-8 secretion compared to wild type cells exposed to the same flagellins (Fig. 8B; p<0.05). These data suggest that knocking down TLR5 significantly decreased IL-8 secretion as stimulated by AP or BL applied flagellin.

## Discussion

It was generally accepted that TLRs were not expressed or expressed at low levels on the AP surface of IEC in order to avoid constitutive intestinal inflammation caused by commensal flora and their products. Based on a study of polarized T-84 IEC it was determined that the flagellin receptor, TLR5, was exclusively location on the BL surface of IEC [33] allowing only Gram-negative pathogens capable of translocating through IEC access to the BL membrane. However, the location of TLR5 on polarized IEC and ability of luminal flagellin to induce innate immune responses is controversial as other investigators have detected TLR5 expression on their luminal surface [35], [36] and shown that flagellin interacts with the AP surface of cultured human IEC to induce pro-inflammatory responses [17], [36], [37]. Our current study concurs with these findings as we report herein that the AP application of flagellin from virulent or avirulent bacteria as well as the flagellin protein constructs of TLR5-binding regions alone stimulated healthy polarized Caco-2BBe and T-84 cells to induce IL-8 secretion and migration of neutrophils and DCs. However, we extend these findings and are the first to show that flagellin is internalized by IEC as directed by apically present TLR5 followed by the co-localization of flagellin with endosomal and lysosomal compartments and subsequently found not to translocate to the BL surface of IEC.

Using a transwell filter system to establish polarized IEC allowed us to expose the AP or BL surface of cells to full-length flagellin from intestinal pathogens or commensal bacteria. Our findings showed that IL-8 was secreted from both the AP and BL surfaces of T-84 and Caco-2BBe cells regardless of which surface was stimulated. However, significantly higher IL-8 secretion was detected in the BL supernatant of both IEC stimulated with full-length flagellins from virulent or avirulent bacteria. These results correlate with the migration of DC and PMN across the IEC barrier. Interestingly, polarized IEC also secreted IL-8 subsequently resulting in the induction of immune cell migration following AP exposure to truncated flagellin protein (ND1/2ECHCD2/1), which contains only the amino and carboxyl conserved flagellin regions. But the hypervariable D3 flagellin region stimulated only minimal to no IL-8 from both cell lines. These findings suggest that flagellin is binding to TLR5, as the conserved regions of this protein are the only domains recognized by TLR5 resulting in the stimulation of host inflammatory responses [15]. In light of these findings, it is not surprising that flagellin from avirulent *E. coli* strain K12 and flagellin from an *E. coli* strain isolated from a Crohn’s disease lesion induced IL-8 secretion and immune cell migration when exposed to the AP surface of IEC. Previous studies have shown that flagellin from a commensal *E. coli* strain induced NF-κB activation via TLR5 binding and the recruitment adaptor protein myeloid differentiation factor-88 (MyD88) in vitro and ex vivo [16].

To determine the role of TLR5 in IEC uptake of flagellin, we suppressed expression of TLR5 by siRNA or blocked TLR5 with an antibody on Caco-2BBe cells then exposed the AP surface to purified flagellin. Both approaches resulted in the detection of AP flagellin but not BL flagellin. Moreover, exposing the siTLR5 cells to purified flagellin significantly decreased IL-8 secretion regardless of AP or BL applied flagellin suggesting that TLR5 is expressed on both surfaces of IEC. These results are in line with other reports that directly detected AP expression of TLR5 on cultured IEC [14], [17], [36], [37] and on human intestinal biopsies [35], [36]. Under certain circumstances live, intact bacteria can influence TLR expression on host cells [38]. However, we did not find that the virulent intestinal pathogen, *S. Typhimurium* and/or an avirulent bacteria, *E. coli* K12, altered TLR5 expression on IEC.

Our findings, as well as others [4], [13], [20], [35]–[37], [39], suggest that the intestinal tract is arranged in such a manner that makes it difficult for resident microflora and their products to contact the AP surface of IEC under steady-state conditions and also has mechanisms in place so that the normal gut does not mount a full-scale immune assault against residential bacteria and their products [40], [41]. Two key factors that limit exposure of the IEC AP surface to commensal bacteria and participate in maintaining intestinal homeostasis is IgA and mucous [18], [40], [42]–[44]. Our recent data indicated that intestinal IgA blocked host systemic immune responses to commensal bacteria [23]. However, pathogenic organisms of the gut have evolved mechanisms (e.g. type III secretion system, exotoxins, endotoxins, use of host proteins as receptors, bacterial effector proteins that manipulate the host cell) that allow them to circumvent host defenses, move through the mucus layer and gain access to IEC [45]–[47]. By breaching these initial defenses, pathogens and their products, such as flagellin, gain access to the AP surface of IEC where specific TLRs provide IEC with the opportunity to detect and respond to pathogens in a timely manner [46]–[47]. Sierro *et al*. [17] showed that *Salmonella* flagellin apically stimulated secretion of CCL20 from IEC resulting in the subsequent migration of DCs. Substantial damage to the luminal surface inflicted by the invading pathogen may also provide an opportunity for commensal flora to access the AP IEC surface [11], [41], [46], [47]. Our studies demonstrate that AP applied flagellin from the commensal *E. coli* K12 strain stimulated IEC leading to IL-8 secretion and immune cell migration. In line with these results, it has been demonstrated that *Salmonella* [48] and Enteroaggregative *E. coli* [39] flagellin as well as flagellin from commensal *E. coli* [16] is capable of inducing inflammatory responses in human IEC.

The intestine has been shown to internalize bacterial products, such as LPS [49], [50] and peptidoglycan [19]. Majority of apically applied purified LPS [22], [51] and/or LPS naturally released from bacteria [52] was internalized by IEC in vitro and in vivo. Our findings showed that a predominant antigen from commensal flora that is found in experimental colitis models known as CBir1 flagellin [23] was internalized by IEC, which could represent potential implications for the contribution of this specific flagellin to Crohn’s disease. Likewise, Caco-2BBe cells took up *Salmonella* flagellin, which was found to co-localized with an early endosomal surface marker and within 1 h flagellin co-localized with a lysosomal membrane surface marker as analyzed by confocal microscopy and cell fractionation. Similarly, it was previously reported that the trafficking of LPS revealed that, following IEC internalization, the highest percentage of LPS co-localized with late endosomes and lysosomes [22]. Our data showed that in regards to cell fractionation of flagellin-exposed cells, the long residence time in the recycling endosome was unexpected. It should be noted that transcytotic traffic was not followed beyond the recycling endosome but the recycling endosome was shared by traffic from both AP and BL surfaces. Therefore, it is possible that residence in the recycling endosome does not preclude traffic across the cell (in either direction).

The biological relevance and significance of our findings demonstrates that luminal recognition of flagellin serves multiple purposes. First, we showed herein that in normal human intestinal epithelial cells, luminal application of flagellin induced innate immune responses that did not disrupt the integrity of the intestinal barrier. Subsequently, IEC internalized flagellin, in a TLR5-dependent manner, which co-localized to early endosomal and lysosomal compartments where, without the addition virulence factors from the live pathogen, it was likely degraded, as it was undetectable on the basolateral side of IEC (i.e. not reaching the immune cell rich lamina propria). These data expand our knowledge of how the normal intestinal epithelial host defense recognition of commensal bacterial products does not elicit a full-scale, unrestrained immune response contributing to intestinal homeostasis. The importance of our findings also lends creditability to emerging evidence that flagellin can be administered under controlled conditions without evoking severe inflammation thus reducing harmful side effects, making it a promising vaccine adjuvant [53].

Second, our data also demonstrate that normal IEC do not distinguish between flagellin monomers of different bacterial species as the amino and carboxyl regions of this protein are conserved among bacterial strains and species. Thus recognition of flagellin, from pathogens such as *S. Typhimurium* and *E. coli* O83:H1 by apically localized TLR5 likely serves as the initial warning against bacterial invaders. Virulence factors possessed by pathogenic bacteria, but absent in commensal flora, are additional contributors to the full-scale, and at times unrestrained, host immune responses. Similar to the lungs, the intestine is constantly exposed to indigenous and environmental bacteria, thus is susceptible to infection. Therefore, it is reasonable to conclude that the host evolved an elaborate, complex immune system to detect dangerous invaders, and their products, at the earliest possible opportunity in order to fight against the infecting pathogens. Mounting evidence shows that the luminal surface of IEC is at the forefront of detecting factors that are injurious to the host, allowing the fight against these harmful factors to begin before they gain access to the lamina propria.

## References

[1] Namba K, Yamashita I, Vonderviszt F (1989). Structure of the core and central channel of bacterial flagella.. Nature.

[2] Eckmann L (2006). Sensor molecules in intestinal innate immunity against bacterial infections.. Current Opin. in Gastroent.

[3] Mobley HLT, Belas R, Lockatell V, Chippendale G, Trifillis AL (1996). Construction of a flagellum-negative mutant of Proteus mirabilis: effect on internalization by human renal epithelial cells and virulence in a mouse model of ascending urinary tract infection.. Infect Immun.

[4] Ohta-Tada U, Takagi A, Koga Y, Kamiya S, Miwa T (1997). Flagellin gene diversity among Helicobacter pylori strains and IL-8 secretion from gastric epithelial cells.. Scand J Gastroenterol.

[5] Penn CW, Luke CJ (1992). Bacterial flagellar diversity and significance in pathogenesis.. FEMS Microbiol Lett.

[6] He X, Rivkina M, Stocker B, Robinson WS (1994). Hypervariable region IV of Salmonella gene fliCd encodes a dominant surface epitope and a stabilizing factor for function flagella.. J Bacteriol.

[7] Mimori-Kiyosue Y, Yamashita I, Yamaguchi S, Namba K (1998). Role of the outermost subdomian of Salmonella flagellin in the filament structure revealed by electron microscopy.. J Mol Biol.

[8] Newton SMC, Wasley RD, Wilson A, Rosenberg LT, Miller JF (1991). Segment IV of a Salmonella flagellin gene specifies flagellar antigen epitopes.. J Mol Microbiol.

[9] Yonekura K, Maki S, Morgan DG, DeRosier DJ, Vonderviszt F (2000). The bacterial flagellar cap as the rotary promoter of flagellin self-assembly.. Science.

[10] Jenal U, Stephens C (2002). The Caulobacter cell cycle: timing, spatial organization and checkpoints.. Curr Opin Microbiol.

[11] Komoriya K, Shibano N, Higano T, Azuma N, Yamaguchi S (1999). Flagellar proteins and type III-exported virulence factors are the predominant proteins secreted into the culture media of Salmonella typhimurium.. Mol Microbiol.

[12] Ramos HC, Rumbo M, Sirard JC (2004). Bacterial flagellins: mediators of pathogenicity and host immune responses in mucosa.. Trends Microbiol.

[13] Eaves-Pyles TD, Wong HR, Odoms K, Pyles RB (2001). Salmonella flagellin-dependent proinflammatory responses are localized to the conserved amino and carboxyl regions of the protein.. J Immunol.

[14] Salazar-Gonzalez RM, McSorley SJ (2005). Salmonella flagellin, a microbial target of the innate and adaptive immune system.. Immunol Lett.

[15] Smith KD, Andersen-Nissen E, Hayashi F, Strobe K, Bergman MA (2003). Toll-like receptor 5 recognizes a conserved site on flagellin required for protofilament formation and bacterial motility.. Nat Immunol.

[16] Bambou JC, Giraud A, Menard S, Begue B, Rakotobe S (2004). In vitro and ex vivo activation of the TLR5 signaling pathway in intestinal epithelial cells by a commensal Escherichia coli strain.. J Biol Chem.

[17] Sierro F, Dubois B, Coste A, Kaiserlian D, Kraehenbuhl JP (2001). Flagellin stimulation of intestinal epithelial cells triggers CCL20-mediated migration of dendritic cells.. Proc Natl Acad Sci U S A.

[18] Chabot SM, Shawi M, Eaves-Pyles T, Neutra MR (2008). Effects of flagellin on the functions of follicle-associated epithelium.. J Infect Dis.

[19] Bu HF, Wang X, Tang Y, Koti V, Tan XD (2010). Toll-like receptor 2-mediated peptidoglycan uptake by immature intestinal epithelial cells from apical side and exosome-associated transcellular transcytosis.. J Cell Physiol.

[20] Eaves-Pyles T, Murthy K, Liaudet L, Virag L, Ross G (2001). Flagellin, a novel mediator of Salmonella-induced epithelial activation and systemic inflammation: IκBα degradation, induction of nitric oxide synthase, induction of proinflammatory mediators, and cardiovascular dysfunction.. J Immunol.

[21] Ibrahim G F, Fleet GH, Lyons MJ, Walker RA (1985). Method for the isolation of highly purified Salmonella flagellin.. J Clin Microbiol.

[22] Beatty WL, Meresse S, Gounon P, Davoust J, Mounier J (1999). Trafficking of Shigella lipopolysaccharide in polarized intestinal epithelial cells.. J Cell Biol.

[23] Cong Y, Feng T, Fujihashi K, Schoeb TR, Elson CO (2009). A dominant, coordinated T regulatory cell-IgA response to the intestinal microbiota.. Proc Natl Acad Sci.

[24] Futter CE, Pearse A, Hewlett LJ, Hopkins CR (1996). Multivesicular endosomes containing internalized EGF-EGF receptor complexes mature and then fuse directly with lysosomes.. J Cell Biol.

[25] Gibson A, Futter CE, Maxwell S, Allchin EH, Shipman M (1998). Sorting Mechanisms Regulating Membrane Protein Traffic in the Apical Transcytotic Pathway of Polarized MDCK Cells.. J Cell Biol.

[26] Norcott JP, Solari R, Cutler DF (1996). Targeting of P-selectin to two regulated secretory organelles in PC12 cells.. J Cell Biol.

[27] Blagoveshchenskaya AD, Cutler DF (2000). Sorting to synaptic-like microvesicles from early and late endosomes requires overlapping but not identical targeting signals.. Mol Biol Cell.

[28] Blagoveshchenskaya AD, Norcott JP, Cutler DF (1998). Lysosomal Targeting of P-selectin Is Mediated by a Novel Sequence within Its Cytoplasmic Tail.. J Biol Chem.

[29] Strasser JE, Arribas M, Blagoveshchenskaya AD, Cutler DF (1999). Secretagogue-triggered Transfer of Membrane Proteins from Neuro-endocrine Secretory Granules to Synaptic-like Micro-vesicles.. Mol Biol Cell.

[30] Wittkop U, Krausse-Opatz B, Gust TC, Kirsch T, Hollweg G (2006). Fate of Chlamydophila pneumoniae in human monocyte-derived dendritic cells: long lasting infection.. Microb Pathog.

[31] Gentry M, Taormina J, Pyles RB, Yeager L, Kirtley M (2007). Role of primary human alveolar epithelial cells in host defense against *Francisella tularensis* infection.. Infect Immun.

[32] Steele-Mortimer O, Meresse S, Gorvel JP, Toh BH, Finlay BB (2005). Biogenesis of Salmonella typhimurium-containing vacuoles in epithelial cells involves interactions with the early endocytic pathway.. Immunol Lett.

[33] Gewirtz AT, Navas TA, Lyons S, Godowski PH, Madara JL (2001). Cutting edge: bacterial flagellin activates basolaterally expressed TLR5 to induce epithelial proinflammatory gene expression.. J Immuol.

[34] Gewirtz AT, Simon PO, Schmitt CK, Taylor LJ, Hagedorn CH (2001). Salmonella typhimurium translocates flagellin across intestinal epithelia, inducing a proinflammatory response.. J Clin Invest.

[35] Cario E, Podolsky DK (2000). Differential alteration in intestinal epithelial cell expression of toll-like receptor 3 (TLR3) and TLR4 in inflammatory bowel disease.. Infect Immun.

[36] Miyamoto Y, Limura M, Kaper JB, Torres AG, Kagnoff MF (2006). Role of Shiga toxin versus H7 flagellin in enterohaemorrhagic Escherichia coli signaling of human colon epithelium in vivo.. Cell Microbiol.

[37] Berin MC, Darfeuille-Michaud A, Egan LJ, Miyamoto Y, Kagnoff MF (2002). Role of EHEC O157:H7 virulence factors in the activation of intestinal epithelial cell NF-kappaB and MAP kinase pathways and the upregulated expression of interleukin 8.. Cell Micro.

[38] Zarember KA, Godowski PJ (2002). Tissue expression of human Toll-like receptors and differential regulation of Toll-like receptor mRNAs in leukocytes in response to microbes, their products, and cytokines.. J Immunol.

[39] Khan MA, Kang J, Steiner TS (2004). Enteroaggregative Escherichia coli flagellin-induced interleukin-8 secretion requires Toll-like receptor 5-dependent p38 MAP kinase activation.. Immunology.

[40] Lu L, Walker WA (2001). Pathologic and physiologic interactions of bacteria with the gastrointestinal epithelium.. Am J Clin Nutr.

[41] Srikanth CV, McCormick BA (2008). Interactions of the intestinal epithelium with the pathogen and the indigenous microbiota: a three-way crosstalk.. Interdiscip Perspect Infect Dis.

[42] Cerutti A (2010). Immunology. IgA changes the rules of memory.. Science.

[43] Cerutti A, Rescigno M (2008). The biology of intestinal immunoglobulin A responses.. Immunity.

[44] Deplancke B, Gaskins HR (2001). Microbial modulation of innate defense: goblet cells and the intestinal mucus layer.. Am J Clin Nutr.

[45] Reading NC, Kasper DL (2011). The starting lineup: key microbial players in intestinal immunity and homeostasis.. Frontiers in Microbiology 2: article.

[46] Artis D (2008). Epithelial-cell recognition of commensal bacteria and maintenance of immune homeostasis in the gut.. Nat Rev Immunol.

[47] Pédron T, Sansonetti P (2008). Commensals, bacterial pathogens and intestinal inflammation: an intriguing ménage à trois.. Cell Host Microbe.

[48] Tallant T, Deb A, Kar N, Lupica J, de Veer MJ, DiDonato JA (2004). Flagellin acting via TLR5 is the major activator of key signaling pathways leading to NF-kappa B and proinflammatory gene program activation in intestinal epithelial cells.. BMC Microbiol.

[49] Harris G, Kuolee R, Chen W (2006). Role of Toll-like receptors in health and diseases of gastrointestinal tract.. World J Gastroenterol.

[50] Hornef MW, Normark BH, Vandewalle A, Normark S (2003). Intracellular recognition of lipopolysaccharide by toll-like receptor 4 in intestinal epithelial cells.. J Exp Med.

[51] Ge Y, Ezzell RM, Warren HS (2000). Localization of endotoxin in the rat intestinal epithelium.. J Infect Dis..

[52] Beatty WL, Sansonetti PJ (1997). Role of lipopolysaccharide in signaling to subepithelial polymorphonuclear leukocytes.. Infect Immun.

[53] Turley CB, Rupp RE, Johnson C, Taylor DN, Wolfson J et al (2011). Safety and immunogenicity of a recombinant M2e-flagellin influenza vaccine (STF2.4xM2e) in healthy adults..

